# The epidemiology of kidney and urinary tract infections in pediatric chronic kidney disease: prevalence, risk factors and association with GFR decline in the Chronic Kidney Disease in Children Study

**DOI:** 10.1007/s00467-026-07253-2

**Published:** 2026-03-25

**Authors:** Derek K. Ng, Andrea R. Molino, Siyao Zhang, Emily J. Stonebrook, Craig S. Wong, Pratyush Gyawali, Kimberly J. Reidy, Susan L. Furth, Bradley A. Warady, Jeffrey J. Fadrowski

**Affiliations:** 1 Department of Epidemiology, Johns Hopkins Bloomberg School of Public Health, 615 N. Wolfe Street Rm. E7642, Baltimore, MD 21075 USA; 2 Department of Epidemiology, University of Washington School of Public Health, Seattle, WA USA; 3 Division of Nephrology and Hypertension, Department of Pediatrics, Nationwide Children’s Hospital, Columbus, OH USA; 4 Department of Pediatrics, Division of Nephrology, University of New Mexico Children’s Hospital, Albuquerque, NM USA; 5 Albert Einstein College of Medicine, Children’s Hospital at Montefiore Einstein, Pediatric Nephrology, Bronx, NY USA; 6 Department of Pediatrics, Division of Nephrology, The Children’s Hospital of Philadelphia, Perelman School of Medicine at the University of Pennsylvania, Philadelphia, PA USA; 7 Department of Pediatrics, Division of Nephrology, Children’s Mercy Kansas City, Kansas City, MO USA; 8 Department of Pediatrics, Division of Nephrology, Johns Hopkins University School of Medicine, Baltimore, MD USA

**Keywords:** Urinary tract infection, Bladder infection, Kidney infection, Congenital anomalies of the kidney and urinary tract, Pediatric chronic kidney disease, Glomerular filtration rate

## Abstract

**Background:**

Urinary tract and kidney infections are potential complications in pediatric chronic kidney disease (CKD), especially among patients with congenital anomalies of the kidney and urinary tract (CAKUT). We characterized the prevalence of self-reported infections from childhood through young adulthood; evaluated risk factors; and quantified the association with CKD progression.

**Methods:**

Infections in the past year were self-reported at annual Chronic Kidney Disease in Children (CKiD) study visits. Age-specific infection prevalences were stratified by sex and CKD diagnoses (glomerular, non-glomerular non-CAKUT, CAKUT low-risk for infections, and three CAKUT high-risk categories: reflux nephropathy, obstructive uropathy, and other high-risk). Repeated measures logistic regression quantified associations between infections and clinical variables. Estimated glomerular filtration rate (eGFR) change in the year after infection was assessed using linear regression, adjusted for age, sex, diagnoses, previous year eGFR, medications, and bladder catheterization.

**Results:**

943 participants contributed 4707 person-visits. Among 175 with obstructive uropathy, approximately one-third of boys and two-thirds of girls reported a previous year infection; infection prevalence for other CAKUT diagnoses ranged from about 20% to 50%. Bladder catheterization and history of infections were significantly associated with recent infections. Adjusted eGFR decline in the year after reporting infection was -1.7% faster compared to those free of infection (95%CI: -3.2%, -0.1%, *p* < 0.05), but this was attenuated when adjusted for catheterization (*p* = 0.082).

**Conclusions:**

CAKUT diagnoses were associated with higher infection risk, especially among those with obstructive uropathy, and girls reported more infections than boys. Self-reported infection was significantly associated with accelerated eGFR decline in the following year.

**Graphical Abstract:**

A higher resolution version of the Graphical abstract is available as Supplementary information.

**Figure:**
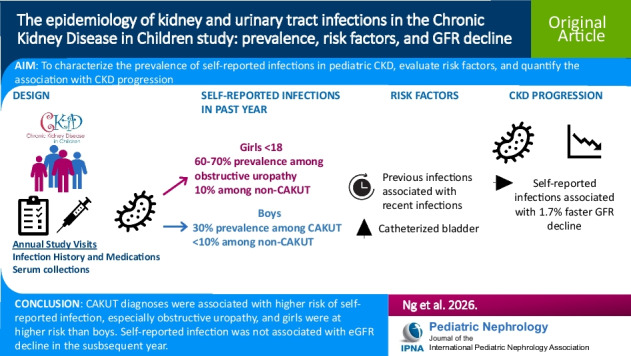

**Supplementary Information:**

The online version contains supplementary material available at 10.1007/s00467-026-07253-2.

## Introduction

Pediatric chronic kidney disease (CKD) is associated with multiple comorbidities including cardiovascular disease, neurocognitive and psychosocial functioning deficits, and short stature [1–3]. Congenital anomalies of the kidneys and urinary tract (CAKUT) are collectively one of the most common causes of CKD in childhood. Urinary tract infections (UTIs) are the most common type of bacterial infection in children and are often an early symptom leading to the diagnosis of CAKUT. Children with CAKUT may have repeated UTIs depending on severity of the anomaly and adequacy of urinary drainage [4, 5]. In addition, therapies used to treat CKD including corticosteroids and other immunosuppressants may increase risk of infection [6, 7]. In the absence of CAKUT, recurrent UTI is not a significant cause of CKD with etiologic fraction < 1%. However, in the presence of CAKUT, it leads to kidney inflammation and scarring, accelerating progression to CKD [8].

In the general pediatric population, UTIs are more common among girls compared to boys [9, 10], and rates of UTIs vary by age [11, 12]. Lower socioeconomic status (SES) has been associated with increased risk of UTIs and kidney infections in the general pediatric population [13]. While recurrent UTI alone is rarely a cause of CKD, UTIs in the presence of vesicoureteral reflux are associated with kidney scarring [8] and lower eGFR [14], and may accelerate CKD progression. In addition, patients with CKD, particularly glomerular diagnoses, may be at increased risk of UTI due to use of corticosteroids and other immunosuppressive therapies. To our knowledge, the epidemiology of self-reported infections of the urinary tract in a large cohort of children and young adults with CKD has not been well described, especially among those with CAKUT diagnoses who are at a putative higher risk of infection, and whether UTIs accelerate GFR decline.

The purpose of this analysis was to: a) describe the prevalence of self-reported annual kidney infections or UTIs (febrile or non-febrile) by CKD diagnoses, sex and age among children, adolescents, and young adults with pediatric CKD, b) investigate putative risk factors associated with self-reported infections, and c) quantify the potential association of self-reported infection on estimated glomerular filtration rate (eGFR) decline in the Chronic Kidney Disease in Children (CKiD) study.

## Methods

### Study design and cohort

The CKiD study is an ongoing, longitudinal cohort of children, adolescents, and young adults with CKD at over 50 sites in the United States and Canada [15, 16]. A total of 1100 participants between the ages of 1 and 16 years of age with mild to moderate CKD were enrolled and completed annual study visits with standard collection of data on kidney disease progression and self-reported infections and other domains through biological specimens and self-administered questionnaires. In this analysis, all participants with CKD were included prior to kidney failure (i.e., post-kidney replacement therapy visits were not included). The CKiD protocol and procedures were approved by the institutional review boards (IRBs) of participating sites, and at a single IRB administered through the Children’s Hospital of Philadelphia (IRB# 10–007880). All participants provided informed consent/assent. The current analysis included data collected from 2006 through 2024.

### Clinical variables

CKiD participants or their caregivers completed self-reported medical history surveys at annual visits, including questions on infections. Participants were asked, “In the past year, has a doctor or any other healthcare professional told you that (participant) had developed any of the following diseases/illnesses?”, with UTI as one of the options (response options: yes or no). Participants were also asked “In the past year, has a healthcare provider diagnosed (participant) with a kidney infection with a fever?” (yes or no). Clinical confirmation by medical records was not available for infection status and this construct of patient-reported infection was the primary outcome. The outcome was summarized as a single composite of any kidney infection or UTI (febrile or non-febrile) in the past year as a binary outcome.

Underlying CKD diagnoses were categorized into six groups: glomerular, non-glomerular (NG) non-CAKUT, NG CAKUT low-risk for infections comprising those with a diagnosis of aplastic/hypoplastic/dysplastic kidneys, and three NG CAKUT high-risk for infections groups — those with a diagnosis of reflux nephropathy, obstructive uropathy, or other high-risk NG CAKUT diagnoses. The other high-risk CAKUT group comprised those with a diagnosis of bilateral hydronephrosis, branchio-oto-renal disease/syndrome, agenesis of abdominal musculature, VACTERL or VATER syndrome, or pyelonephritis/interstitial nephritis. A full description and distribution of the diagnoses comprising these broad categories is presented in Supplemental Table 1.

Age was stratified as < 12, ≥ 12 to 18, and ≥ 18 years. Race/ethnicity was defined by self- or parental-reported race and Hispanic ethnicity. Additional self-reported SES variables included time-varying annual income (> $75 k vs. $36 k to $75 k vs. < $36 k), baseline maternal education (college or more vs. less than college), and time-varying health insurance status (public vs. private).

CKD characteristics included eGFR, urine protein/creatinine ratio (uPCR; mg/mgCr), proteinuria defined as normal (< 0.2 mg/mgCr), elevated (0.2 to < 2 mg/mgCr), or nephrotic range (≥ 2 mg/mgCr), and duration since CKD diagnosis. eGFR was primarily based on the cystatin C Under 25 equation [17] because serum creatinine can be modified by trimethoprim without affecting underlying kidney function [18]. To minimize missing data, if cystatin was not available, SCr-based U25eGFR was used. Additional clinical variables included blood pressure stage (normal vs. elevated, stage 1 or 2 hypertension) [19], anemia (yes vs. no) [20], past 30-day use of immunosuppressant therapy, corticosteroid therapy, any antibiotics, TMP-SMX (trimethoprim-sulfamethoxazole/Bactrim), diuretics, and bladder medications. We also investigated experience of wetness or leakage of urine (i.e., urinary incontinence) during the day or night in the past year. Among those with a CAKUT diagnosis, we also included bladder catheterization which was expected to be associated with a higher risk of infection.

### Statistical analysis

To estimate the prevalence of past year composite infection (UTI or kidney) for each CKD diagnosis and age group combination, we used repeated measures logistic regression models (with generalized estimating equations [GEE], robust standard errors, and compound symmetry covariance structures) for each sex, using person-visits as the unit of analysis. Diagnosis- and age-specific prevalences with 95% confidence intervals were estimated for glomerular, NG non-CAKUT, CAKUT low-risk, and the three CAKUT high-risk groups and age < 12, 12 to < 18, and ≥ 18 years.

To identify putative risk factors, separate repeated measures logistic regression models with GEE were fit for each risk factor and CKD diagnosis group. The outcome was past year infection, and the independent variable was the risk factor of interest, with adjustment for sex and age (continuous). Risk factors included race, ethnicity, income, baseline maternal education, health insurance, categorical eGFR, categorical proteinuria, elevated blood pressure/hypertension, anemia, past 30-day medication use (immunosuppressant therapy, corticosteroid therapy, any antibiotics including TMP-SMX, diuretics, and bladder medications), reported past year UTI/kidney infection from the most recently available previous visit, reported urinary incontinence (accidents) in the past year, and catheterization of the bladder in the past year. Importantly, all risk factor variables were lagged in models so that the risk factor data was prior to the reporting of the past year infection, ensuring temporality and limiting the risk of reverse causation. Missing data was minimal (Supplemental Table 2) and analyses used a complete case approach.

To quantify the association between infections and eGFR decline, repeated measures linear regression models with GEE were used. The dependent variable was annualized percent change in eGFR, calculated as log-transformed eGFR at the present visit minus log-transformed eGFR at the previous visit divided by the time in years elapsed between the two measurements [21]. The independent variable was past year infection. Diagnosis-specific models, and models pooling diagnoses, were fit with adjustment for sex, continuous age, previous visit eGFR (log-transformed), and previous year (lagged) use of antibiotics and bladder medications, and additional models were further adjusted for bladder catheterization at the previous visit to assess its role as a potential confounder in the relationship between infection in the past year and eGFR.

Statistical significance was assessed at *p* < 0.05. For the risk-factor analysis, correction for multiple comparisons was based on the Holms method with a family wise error rate of 10% (FWER *p* = 0.10). All analyses were performed in SAS 9.4 (SAS Institute Inc., Cary, NC), and figures were created using R 4.0.2 (Vienna, Austria).

## Results

Table 1 shows demographic and clinical characteristics of our study population at analytical baseline, stratified by CKD diagnosis groups (see Supplemental Table 1 for specific diagnoses). Overall, 239 (24.6%) participants with glomerular diagnoses, 166 (17.1%) NG non-CAKUT, 184 (18.9%) NG CAKUT low risk, 128 (13.2%), 175 (18.0%) NG CAKUT high-risk (reflux nephropathy, obstructive uropathy), and 79 (8.1%) had other CAKUT high-risk. Identification of kidney problems during pregnancy was most common among those with CAKUT high-risk (51%), CAKUT low-risk (43%), and obstructive uropathy (41%). At baseline, compared to the NG groups, those with glomerular disease were older (median = 15 years [IQR: 12, 17] vs. 8 to 11 years), with shorter disease duration (median = 4.7 [2.5, 8.6] vs. 8 to 11 years) and with higher eGFR (median = 56 [37, 75] vs. 45 to 53 mL/min/1.73 m^2^, and cystatin C-based eGFR data were available for approximately 90% of person-visits). They were more likely to have nephrotic range proteinuria (19% vs. 3 to 9%) and were more likely to report immunosuppressant therapy use (44% vs. 1 to 4%). Compared with the NG non-CAKUT and CAKUT low-risk, those with obstructive uropathy and other NG CAKUT high-risk diagnosis were more likely to be male (82 to 89% vs. 52 to 59%), report any antibiotic use (25 to 48% vs. 9 to 18%), and report bladder catheter usage (33 to 44% vs. 2 to 20%). In addition, compared to all other groups, those with obstructive uropathy were more likely to report TMP-SMX use (25% vs. 4 to 14%) and bladder medication (26% vs. 1 to 11%).

**Table 1:** Baseline characteristics of 971 participants, stratified by underlying CKD diagnosis

	Glomerular *n* = 239	NG non-CAKUT *n* = 166	NG CAKUT low-risk *n* = 184	Reflux nephropathy *n* = 128	Obstructive uropathy *n* = 175	NG CAKUT high-risk other *n* = 79
**Sociodemographic characteristics**						
Age, years	15.1 [11.9, 16.9]	9.7 [6.1, 14.0]	8 [4.9, 12.4]	11.4 [7, 15.1]	8.9 [5.6, 13.2]	9.2 [5.1, 14.2]
Male sex	52% (123)	54% (90)	59% (109)	57% (73)	89% (155)	82% (65)
Self-reported Black race	31% (73)	11% (19)	19% (35)	6% (8)	33% (57)	20% (16)
Non-White and/or Hispanic ethnicity	56% (133)	32% (53)	43% (79)	30% (39)	47% (83)	39% (31)
Annual income						
> $75 k	33% (77)	37% (61)	35% (63)	41% (52)	29% (50)	38% (29)
$36 k to $75 k	27% (63)	32% (53)	25% (44)	28% (35)	31% (54)	22% (17)
≤ $36 k	41% (96)	31% (51)	40% (72)	31% (40)	41% (71)	40% (31)
Maternal education: less than college	67% (158)	57% (95)	65% (119)	63% (79)	68% (119)	61% (48)
Health insurance type						
Private	47% (107)	51% (83)	46% (84)	59% (72)	43% (73)	53% (42)
Public	52% (118)	49% (81)	51% (92)	36% (44)	55% (94)	47% (37)
**Kidney and clinical characteristics**						
Identification of kidney abnormality						
During pregnancy	1% (2)	19% (31)	43% (79)	25% (32)	41% (71)	51% (40)
CKD duration, years	4.7 [2.5, 8.6]	8.2 [4.6, 11.3]	8 [4.9, 12.4]	11.2 [6.9, 15]	8.9 [5.6, 13.2]	7.9 [5, 12.3]
eGFR, ml/min|1.73 m^2^	56 [37, 75]	46 [31, 61]	45 [32, 59]	51 [34, 62]	48 [34, 61]	53 [39, 67]
Urine protein/creatinine, mg/mgCr	0.5 [0.2, 1.7]	0.2 [0.1, 0.6]	0.3 [0.1, 0.9]	0.2 [0.1, 0.6]	0.4 [0.1, 0.9]	0.3 [0.1, 0.9]
Proteinuria						
Normal (< 0.2 mg/mgCr)	31% (71)	50% (72)	34% (55)	50% (61)	33% (51)	43% (30)
Elevated (0.2 to 2 mg/mgCr)	50% (114)	41% (60)	57% (91)	47% (57)	58% (88)	49% (34)
Nephrotic range (≥ 2 mg/mgCr)	19% (43)	9% (13)	9% (14)	3% (4)	9% (14)	9% (6)
Elevated blood pressure/hypertension	39% (92)	34% (53)	43% (74)	30% (37)	48% (80)	40% (30)
Anemia	38% (88)	30% (49)	17% (31)	24% (29)	21% (36)	16% (12)
Past 30-day medication use						
Immunosuppressant therapy^a^	44% (105)	2% (3)	1% (1)	2% (2)	1% (1)	4% (3)
Corticosteroid therapy	21% (49)	2% (3)	1% (1)	2% (2)	0% (0)	4% (3)
Antibiotics	13% (30)	9% (15)	18% (33)	25% (32)	48% (83)	34% (27)
TMP-SMX	5% (12)	4% (6)	9% (17)	13% (17)	25% (44)	14% (11)
Diuretics	13% (30)	11% (18)	3% (5)	1% (1)	1% (2)	1% (1)
Bladder medication	1% (3)	4% (6)	8% (15)	7% (9)	26% (45)	11% (9)
Enuresis (incontinence) during the day or night	19% (40)	57% (83)	60% (97)	38% (39)	69% (103)	48% (33)
Catheterized bladder	2% (4)	4% (6)	17% (28)	20% (21)	44% (65)	33% (23)
Longitudinal data						
Person-Visits	1010	855	961	635	360	886

^a^Including corticosteroid therapy

Presented as median [IQR] or percent (N). *NG* non-glomerular, *CAKUT* congenital anomalies of kidney and urinary tract

**Fig. 1:**
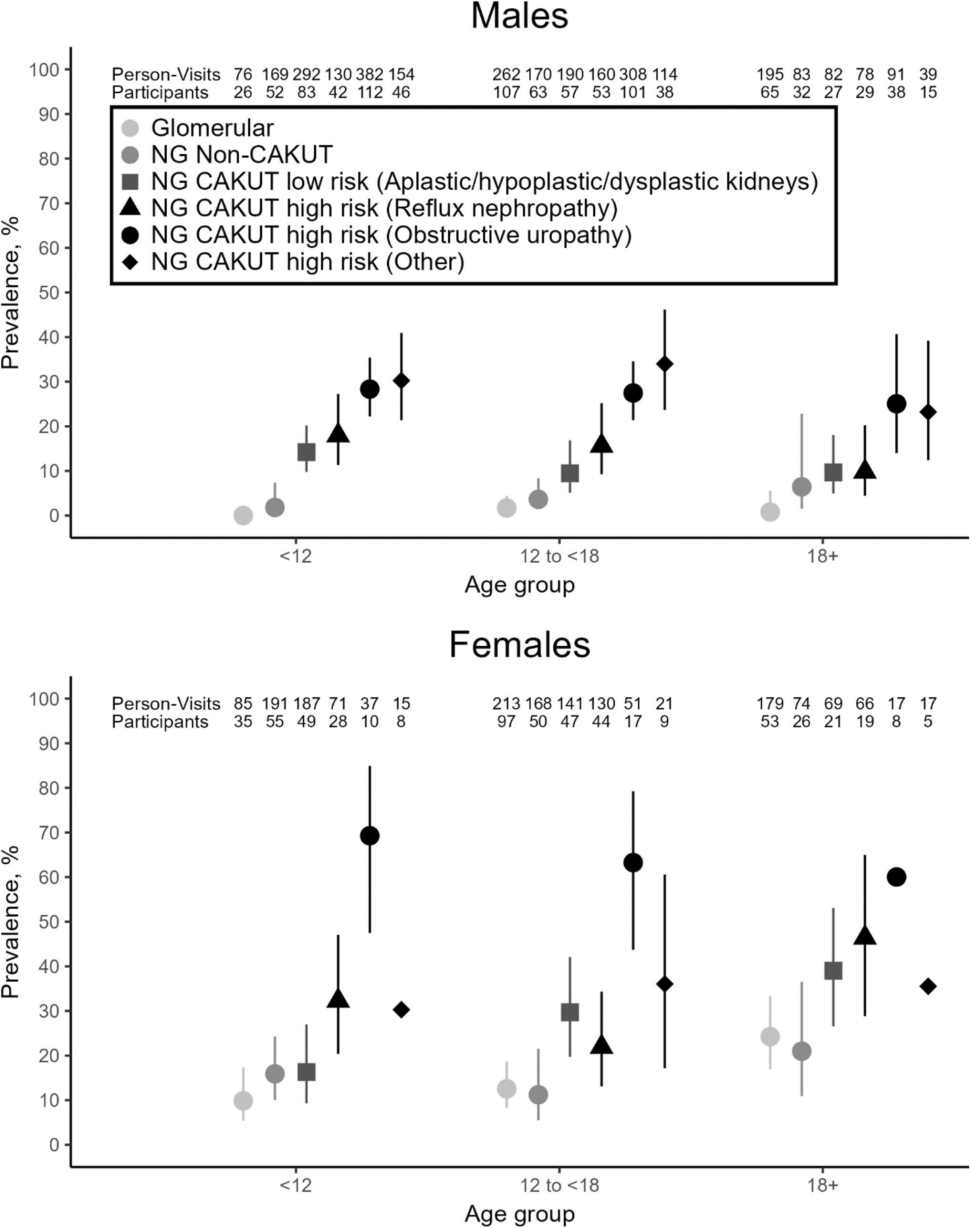
Estimated prevalence and 95% confidence intervals of past year infection, stratified by sex, age category, and primary CKD diagnosis. Person-visits and individuals contributing to the corresponding data point are shown in the first and second row across the top, respectively

Across all visits, 17.5% reported any infection (UTI or kidney infection). Of those who reported UTI, 43.6% also reported a kidney infection; 6.5% (= 53/812) of those who reported any infection only reported a kidney infection with fever without a UTI (Supplemental Table 2). For the composite of any self-reported infection, Fig. 1 displays the prevalence of past year UTIs/kidney infections, stratified by sex, age category, and primary CKD diagnosis. In both sexes and in all age groups, prevalence of infection was lowest among those with glomerular diagnoses, and highest among those with NG CAKUT high-risk. Risk of infection in boys with glomerular disease was overall low: with the highest prevalence estimate at 1.7% (95%CI: 0.6%, 4.4%) among those 12 to < 18 years, and less than 0.1% reported infections among those < 12 years. Conversely, there was a lower infection prevalence with older age among boys with NG CAKUT low-risk diagnoses, and a consistent higher prevalence between 23 to 34% among boys with obstructive uropathy and other NG CAKUT high-risk participants.

Among girls, the prevalence of past year infections was highest among the oldest age range except for those with obstructive uropathy. About two-thirds of girls with obstructive uropathy diagnoses reported an infection in the past year for those < 12 years (69%, 95%CI: 48%, 85%) and 12 to < 18-years (63%, 95%CI: 44%, 79%), and older than 18 (60%, 95%CI: 38%, 79%). For other NG CAKUT high-risk diagnoses, the highest prevalence was reported for those from 12 to < 18 years (36%, 95%CI: 17%, 61%). For other diagnoses, the highest burden was experienced among girls ≥ 18 years: estimated prevalence for those with glomerular, NG non-CAKUT, and NG CAKUT low-risk diagnoses were 24% (95%CI: 17%, 33%), 21% (95%CI: 11%, 37%), and 39% (95%CI: 27%, 53%), respectively.

Table 2 presents results from models exploring potential risk factors associated with infections, controlling for age and sex, and with Holm’s p-value adjustment for multiple comparisons. Previous infections were most consistently associated with a higher prevalence odds of current infection, particularly for the NG non-CAKUT group (OR: 23.6; 95%CI: 9.65, 57.7), CAKUT low-risk (OR: 4.19, 95%CI: 2.27, 7.75), obstructive uropathy (OR: 2.49, 95%CI: 1.50, 4.15), and other CAKUT high-risk (OR: 6.13, 95%CI: 2.73, 13.8). Bladder catheterization was associated with past year infection for all NG CAKUT high-risk groups. Lastly, enuresis (incontinence) during the day or night was associated with infections in participants with reflux nephropathy (2.38, 95%CI: 1.49, 3.81), but not with other diagnosis categories.

**Table 2:** Quantifying potential risk factors for infections by repeated measures logistic regression models stratified by primary diagnosis showing the odds ratio (95% confidence intervals) of reported past year kidney infection or UTI, adjusted for sex and continuous age

	Glomerular, girls^a^	NG non-CAKUT	NG CAKUT low-risk	Reflux nephropathy	Obstructive uropathy	NG CAKUT high-risk other
**Person-visits**	**477**	**855**	**961**	**635**	**886**	**360**
Black race	1.14 (0.51, 2.55)	1.10 (0.38, 3.25)	1.00 (0.44, 2.31)	1.29 (0.34, 4.91)	1.04 (0.63, 1.72)	1.02 (0.36, 2.89)
Non-White race and/or Hispanic ethnicity	1.19 (0.59, 2.37)	1.15 (0.47, 2.82)	1.31 (0.73, 2.35)	0.89 (0.43, 1.84)	1.14 (0.71, 1.85)	1.36 (0.62, 2.97)
Annual household income						
> $75,000	REF	REF	REF	REF	REF	REF
$36,000 to $75,000	0.70 (0.38, 1.31)	0.56 (0.21, 1.49)	0.77 (0.45, 1.31)	1.12 (0.65, 1.92)	0.74 (0.51, 1.07)	1.70 (0.79, 3.66)
≤ $36,000	0.91 (0.45, 1.85)	1.05 (0.44, 2.47)	1.43 (0.86, 2.37)	1.63 (0.98, 2.72)	1.09 (0.72, 1.65)	*2.54 (1.25, 5.16)*
Maternal education: less than college^b^	1.02 (0.47, 2.19)	1.12 (0.46, 2.72)	1.60 (0.83, 3.11)	1.72 (0.84, 3.53)	0.98 (0.59, 1.64)	1.81 (0.82, 3.96)
Public health insurance	1.28 (0.70, 2.34)	1.68 (0.92, 3.09)	1.28 (0.80, 2.06)	1.04 (0.65, 1.68)	0.93 (0.62, 1.38)	1.31 (0.80, 2.14)
eGFR category^c^						
> 60 ml/min|1.73 m^2^	REF	REF	REF	REF	REF	REF
45 to 60 ml/min/1.73 m^2^	0.89 (0.45, 1.75)	1.05 (0.46, 2.41)	0.76 (0.45, 1.31)	0.99 (0.59, 1.63)	1.30 (0.77, 2.19)	1.61 (0.93, 2.77)
30 to 45 ml/min/1.73 m^2^	0.45 (0.18, 1.12)	1.21 (0.57, 2.57)	1.14 (0.65, 2.00)	0.79 (0.43, 1.42)	1.63 (0.97, 2.74)	1.37 (0.68, 2.74)
≤ 30 ml/min/1.73 m^2^	1.33 (0.55, 3.23)	1.10 (0.48, 2.54)	1.12 (0.56, 2.27)	0.69 (0.34, 1.40)	1.29 (0.70, 2.36)	*2.14 (1.00, 4.61)*
Proteinuria^c^						
Normal (< 0.2 mg/mgCr)	REF	REF	REF	REF	REF	REF
Elevated (0.2 to 2 mg/mgCr)	1.18 (0.63, 2.20)	1.16 (0.77, 1.75)	*1.78 (1.04, 3.05)*	1.03 (0.65, 1.64)	1.35 (0.93, 1.95)	1.46 (0.89, 2.38)
Nephrotic range (≥ 2 mg/mgCr)	1.33 (0.55, 3.23)	0.97 (0.37, 2.59)	1.57 (0.68, 3.63)	0.89 (0.35, 2.26)	1.28 (0.68, 2.43)	1.57 (0.63, 3.90)
Elevated blood pressure/hypertension^c^	0.96 (0.54, 1.72)	1.00 (0.65, 1.54)	1.24 (0.84, 1.84)	1.04 (0.70, 1.56)	1.16 (0.87, 1.55)	1.30 (0.84, 1.99)
Anemia^c^	1.01 (0.57, 1.79)	1.16 (0.75, 1.78)	1.01 (0.58, 1.74)	0.74 (0.45, 1.20)	1.04 (0.72, 1.51)	1.16 (0.70, 1.92)
Medication use^c^						
Immunosuppressant therapy^d^	*2.16 (1.18, 3.96)*	1.20 (0.36, 4.03)	Not estimated	Not estimated	Not estimated	Not estimated
Corticosteroid therapy	1.79 (0.95, 3.35)	1.27 (0.32, 5.02)	Not estimated	Not estimated	Not estimated	Not estimated
Antibiotics	0.80 (0.35, 1.82)	1.91 (0.65, 5.56)	1.24 (0.65, 2.37)	*1.87 (1.10, 3.18)*	1.41 (0.93, 2.15)	*2.34 (1.32, 4.13)*
TMP-SMX	0.92 (0.35, 2.38)	1.31 (0.29, 5.96)	1.19 (0.44, 3.21)	0.87 (0.38, 2.02)	1.01 (0.66, 1.56)	1.45 (0.47, 4.52)
Diuretics	0.43 (0.16, 1.18)	0.53 (0.12, 2.23)	1.73 (0.53, 5.72)	Not estimated	Not estimated	Not estimated
Bladder medication	2.21 (0.28, 17.4)	2.46 (0.63, 9.64)	1.12 (0.36, 3.43)	*2.98 (1.60, 5.53)*	1.55 (0.97, 2.47)	*3.09 (1.22, 7.82)*
Report of past year infection at previous visit^c^	*3.49 (1.34, 9.11)*	***23.6 (9.65, 57.7)***	***4.19 (2.27, 7.75)***	1.40 (0.68, 2.90)	***2.49 (1.50, 4.15)***	***6.13 (2.73, 13.8)***
Enuresis (incontinence) during the day or night	1.63 (0.74, 3.58)	1.89 (0.96, 3.72)	0.96 (0.57, 1.60)	***2.38 (1.49, 3.81)***	0.81 (0.56, 1.16)	1.23 (0.61, 2.50)
Catheterized bladder^c^	1.39 (0.13, 15.0)	*4.03 (1.21, 13.4)*	2.09 (0.99, 4.38)	***6.84 (3.50, 13.4)***	***4.70 (2.95, 7.47)***	***4.15 (2.03, 8.50)***

^a^Glomerular models only included girls because the prevalence of infection among boys with glomerular disease was < 1% (= 5/527 person-visits)

^b^Baseline maternal education used

^c^Risk factors were lagged, defined from the most recent visit within two years before the self-report outcome of infection in the past year

^d^Including corticosteroid therapy

*NG* non-glomerular, *CAKUT* congenital anomalies of kidney and urinary tract. Italicized values denote unadjusted significant *p*-values (< 0.05); **bold** and italicized values denote *p* < 0.1 after adjustment by Holm’s method. Results are not estimated for groups with <  = 3 person-visits for the exposed risk factor. Boys with glomerular diagnoses were not included because of low prevalence of infections. Missing data is described in Supplemental Table 2

**Fig. 2:**
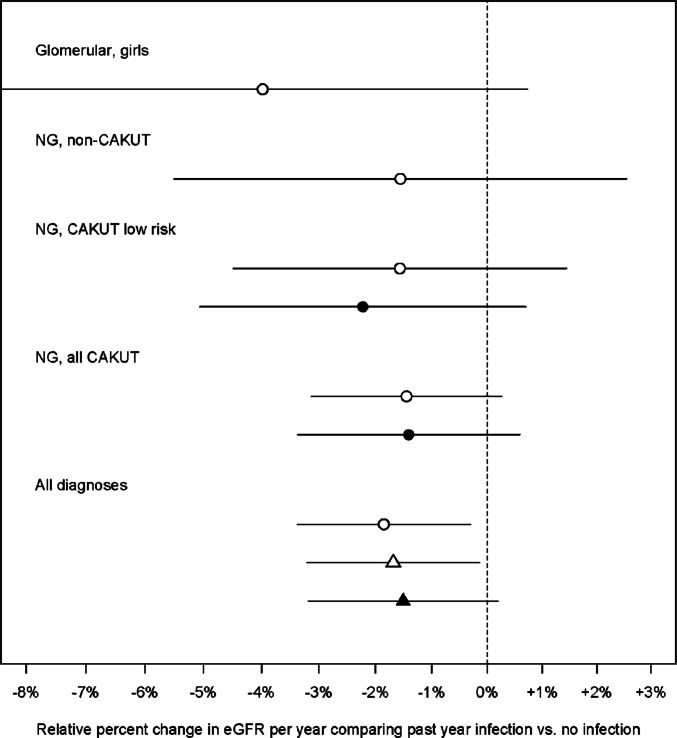
Relative annualized percent change in eGFR and 95% confidence intervals comparing those who reported infection to those who did not report infection in the past year from repeated measures linear regression. Open circles (◯) show results from the base model (adjusting for sex, continuous age, previous visit eGFR log-transformed, and lagged use of antibiotics and bladder medications). Solid circles (●) show results from the base model plus additional adjustment for report of past year bladder catheterization at the previous visit (only relevant for those with CAKUT diagnoses). Empty triangles (△) show results from the base model for all diagnoses plus adjustment for CKD diagnosis (glomerular, NG non-CAKUT, NG CAKUT low-risk, three NG CAKUT high-risk groups). Solid triangles (▲) show results from the base model for all diagnoses plus adjustment for the ten-level diagnosis/catheterization combination variable (glomerular, NG non-CAKUT, NG CAKUT low-risk with no catheterization report at previous visit, NG CAKUT low-risk with catheterization, and the three NG CAKUT high-risk groups with and without catheterization). Models of the three NG-CAKUT high-risk groups were explored, but the differences were not significant and the inferences were the same as the pooled all CAKUT diagnoses

Figure 2 presents results from models quantifying relative annualized eGFR change, comparing those who did vs. did not report an infection in the past year, with adjustment for confounders. Among those who reported past year infection, eGFR declined between 0.8% to 4.0% per year faster compared to those who did not report infection, but were not significant in diagnosis specific-models. When pooling all diagnoses with full adjustment for age, sex, previous eGFR, therapies, the relative annualized eGFR decline for those reporting past year infection was significantly faster by −1.7% (95%CI: −3.2%, −0.1%) compared to no infection. After adjustment for catheterization, which may be an indicator for chronic, recurrent infection risk, the estimate was about the same, but with slightly less precision: −1.5% (95%CI: −3.2%, + 0.2%, p = 0.082) and was borderline significant. In sensitivity analyses, we did not observe that self-reported kidney infection was associated with accelerated GFR decline compared to self-reported UTI.

## Discussion

In this population of children and young adults with pediatric CKD, we found a substantial burden of patient-reported urinary tract/kidney infections. Moreover, infections were not uniform across sex and diagnoses. Within diagnoses, girls were at much higher risk than boys: about two-thirds of girls with CAKUT diagnoses reported having had an infection in the past year compared to one-third of boys. The highest risk was observed among girls with high-risk CAKUT diagnoses comprising obstructive uropathy, agenesis of abdominal musculature, VACTERL or VATER syndrome, or pyelonephritis/interstitial nephritis, and the prevalence rate was consistently around 60% across age groups. Among boys, the highest infection rates were also observed in those with CAKUT high-risk diagnoses, with the prevalence consistently around 30% across ages. Boys were much more likely to have CAKUT high-risk diagnoses compared to girls (88% vs. 12%). However, among those with CAKUT high-risk diagnoses, girls reported infections at nearly twice the rate of boys (~ 60% vs. ~ 30%).

Previous studies reported a higher burden of infections among those with obstructive uropathies (e.g., CAKUT HR group) [22–24]. While it is perhaps not surprising that the structural pathologies associated with CAKUT diagnoses were associated with higher rates of infection, to our knowledge, this study is the first to quantify this burden by diagnosis, age and sex, especially highlighting the excess burden among girls generally, and specifically, girls with obstructive uropathy. While we were not able to present diagnosis-specific infection rates, the grouping of diagnoses was determined a priori based on structural pathologies and the results demonstrated the expected directionality. Glomerular diagnoses were associated with the lowest risk, and these general groupings can help guide clinicians for patient and family counseling, as well as prompt treatment and potential prophylactic regimens.

Antibiotic prophylaxis in VUR as demonstrated in both the RIVUR study and PREDICT studies were associated with decreased risk of recurrent UTI [25–29]. Despite these known benefits of prophylactic antibiotic use, infections in the CAKUT high-risk group remained high. Interestingly, TMP-SMX antibiotic prophylaxis was not statistically associated with decreased UTI risk, although the increasing prevalence of TMP-SMX usage by diagnostic group reflected the increasing infection risk (e.g., 5% for those with glomerular diagnoses, 11% for those with CAKUT low-risk and 26% for those with CAKUT high-risk). The lack of association and reduced infection risk might also be related to dosage, adherence or antimicrobial resistance. Further investigation into the real-world effectiveness of antibiotic prophylaxis is needed.

The two strongest risk factors for infections included an infection in the past year and use of a bladder catheter. The high ORs associated with infections in the past year reflects the chronic and recurrent nature of infections in this population. Regarding bladder catheterization, significant confounding by indication likely underlies this association as this invasive intervention is reserved for those with the worst urinary drainage, most often represented by increased frequency of UTI. It was reassuring that other variables including CKD severity (i.e., GFR and proteinuria) were not associated with increased risk of infection. Low income was associated with a higher risk of infection in the CAKUT low-risk group, but other socioeconomic variables were not, and the lack of consistency may suggest it is an artifact of the data.

In prospective analyses, there was a consistent pattern that the presence of infection was associated with approximately a 2% faster GFR decline over a one-year period. The exact mechanism of this is unclear but it could be related to increased inflammation/injury of the kidney secondary to infection, and for some, potentially inadequate urinary bladder drainage. In adjusted analyses, this association appeared to be at least partially mediated by bladder catheterization. This finding is congruent with a single center cross-sectional study of 405 children and adolescents with CAKUT that showed UTI as the presenting symptom was associated with higher CKD stage [30] and reports of lower GFR in children with VUR and UTIs [14].

This analysis did not demonstrate a strong decreasing association of UTI risk with age. Indeed, the prevalence of infection among boys with high-risk NG diagnoses remained constant across all ages, and the prevalence increased across ages among all girls in the cohort. More data are needed to address the hypothesis that infection risk declines with age because behavioral risk factors change across ages. In addition, further study of the effect of antibiotic prophylaxis in children with CAKUT in real world settings is warranted.

There were several limitations to this study. Data were not available on severity and number of infection: the UTI question did not distinguish between febrile or non-febrile infections, and the kidney infection question did not include non-febrile infection. Diagnoses were reduced to a primary cause of CKD, did not consider multiple phenotypes and were broadly grouped into categories. Heterogeneity in obstructive uropathy diagnoses with differential risk such as ureterovesical junction obstruction (UVJO; higher risk) and posterior urethral valves (PUV; higher risk), and ureteropelvic junction obstruction (UPJO; lower risk) were not available, although efforts are underway to refine the diagnosis data within CKiD. Among patients with reflux nephropathy, it is noted that 30/128 (39%) reported enuresis/incontinence and 20% reported bladder catheterization; both factors were associated with UTI in this group. It is possible that there was misclassification of the underlying CKD diagnosis with the urinary reflux being secondary rather than primary, for example due to dysfunctional elimination syndrome, a PUV, or other diagnosis associated with bladder dysfunction. In addition, data was not collected on dosing, timing and duration of antibiotic use. Hospitalization data was collected [31], but did not include the reason, which may or may not have been related to infection. Lastly, we could not validate the self-reported infections with clinical confirmation or identify current infection risk based on the data. Misclassification of self-reported infections was possible, but it is most likely an underestimate of UTIs with the most severe infections recalled by participants.

In summary, this study described the high burden of UTIs and kidney infections across sex and age among individuals with pediatric CKD, particularly those with obstructive uropathy, agenesis of abdominal musculature, and VACTERL or VATER syndrome. Catheterization and a history of a UTI or kidney infection in the last year were major risk factors, but no other measured clinical risk factors were consistently identified. Lastly, there was an indication of an accelerated annual decline in GFR associated with a history of infection, which is likely clinically important, although this was slightly attenuated when adjusting for catheterization. Given the high burden of infections, future work could further characterize the impact of UTIs and kidney infections on long term GFR decline in addition to investigating how UTIs impact healthcare utilization, including hospitalizations, and quality of life in this population [32]. In the interim, clinicians should be aware of the increased risk of UTI in certain groups of children with CKD and be vigilant in screening for and treating UTI in those with early symptoms to mitigate the many known and potential adverse impacts of this common morbidity.

## Supplementary Information

Below is the link to the electronic supplementary material.

Supplementary file1 Graphical abstract (PPTX 183 KB)

Supplementary file2 (DOCX 28 KB)

## Data Availability

Data from The Chronic Kidney Disease in Children Cohort Study (CKiD) [(Version 8) 10.58020/dzq8-ct80] are available for request at the NIDDK Central Repository (NIDDK-CR) website, Resources for Research (R4R), https://repository.niddk.nih.gov/.
