# Supplementary material for: The epidemiology of kidney and urinary tract infections in pediatric chronic kidney disease: prevalence, risk factors and association with GFR decline in the Chronic Kidney Disease in Children Study
Source: Pediatr Nephrol. 2026 Mar 25;41(10):3377–87. doi: 10.1007/s00467-026-07253-2 (PMC13481756; doi:10.1007/s00467-026-07253-2)
Supplement: Supplementary file 1 [file 467_2026_7253_MOESM2_ESM.docx]

**Supplemental Material**

**Supplemental Table 1.** Classification and distribution of analytical diagnosis categories for 971 participants in study population. NG= non-glomerular; CAKUT= congenital anomalies of kidney and urinary tract.

**Supplemental Table 2.** Distribution of self-reported urinary tract infections and self-reported kidney infections with fever among all person-visits (n= 4633).

**Supplemental Table 3.** Characterization of missing data, stratified by diagnosis.

**Supplemental Table 4.** List of principal site investigators of the Chronic Kidney Disease in Children (CKiD) cohort study.

**Supplemental Table 1.** Classification and distribution of analytical diagnosis categories for 971 participants in study population. NG= non-glomerular; CAKUT= congenital anomalies of kidney and urinary tract.

| **Glomerular**  **n= 239 (24.6%)** | | **NG non-CAKUT**  **n= 166 (17.1%)** | | **NG CAKUT low-risk**  **n= 369 (38.0%)** | | **NG CAKUT high-risk**  **n= 197 (20.3%)** | |
| --- | --- | --- | --- | --- | --- | --- | --- |
| Focal segmental glomerulosclerosis (FSGS) | 28% (67) | Non-Glomerular Other | 26.5% (44) | Aplastic/hypoplastic/dysplastic kidneys | 100% (184) | Obstructive uropathy | 45.8% (175) |
| Hemolytic uremic syndrome | 20.1% (48) | Polycystic kidney disease (Autosomal recessive) | 21.7% (36) |  |  | Reflux nephropathy | 33.5% (128) |
| Systemic immunological disease (including SLE) | 14.2% (34) | Renal infarct | 14.5% (24) |  |  | Congenital Urologic Disease (Bilateral Hydronephrosis) | 12.8% (49) |
| Chronic glomerulonephritis | 7.5% (18) | Cystinosis | 7.8% (13) |  |  | Syndrome of agenesis of abdominal musculature | 2.4% (9) |
| Familial nephritis (Alport's) | 5.9% (14) | Perinatal Asphyxia | 7.2% (12) |  |  | Branchio-oto-Renal Disease/Syndrom | 2.1% (8) |
| IgA Nephropathy (Berger's) | 5.9% (14) | Medullary cystic disease/Juvenile nephronophthisis | 5.4% (9) |  |  | VACTERL or VATER Syndrome | 2.1% (8) |
| Membranoproliferative glomerulonephritis type I | 5% (12) | Wilms' tumor | 4.2% (7) |  |  | Pyelonephritis/interstitial nephritis | 1.3% (5) |
| Glomerular Other | 3.3% (8) | Interstitial nephritis | 4.2% (7) |  |  |  |  |
| Henoch schonlein nephritis | 2.9% (7) | Methylmalonic Acidemia | 3.6% (6) |  |  |  |  |
| Idiopathic cresentic glomerulonephritis | 2.5% (6) | Polycystic kidney disease (Autosomal dominant) | 3.6% (6) |  |  |  |  |
| Congenital nephrotic syndrome | 1.7% (4) | Oxalosis | 1.2% (2) |  |  |  |  |
| Membranous nephropathy | 1.7% (4) |  |  |  |  |  |  |
| Membranoproliferative glomerulonephritis type II | 0.8% (2) |  |  |  |  |  |  |
| Sickle cell nephropathy | 0.4% (1) |  |  |  |  |  |  |

**Supplemental Table 2.** Distribution of self-reported urinary tract infections and self-reported kidney infections with fever among all person-visits (n= 4633 person-visits from 943 CKiD participants).

|  | **No self-reported UTI** | **Self-reported UTI** | **Total** |
| --- | --- | --- | --- |
| **No self-reported kidney infection w/ fever** | 3821 (82%) | 428 (9%) | 4249 (92%) |
| **Self-reported kidney infection w/ fever** | 53 (1%) | 331 (7%) | 384 (8%) |
| **Total** | 3874 | 759 | 4633 |

Missing data: self-reported UTI n= 29; self-reported kidney infection with fever n= 45.

**Supplemental Table 3.** Characterization of missing data, stratified by diagnosis.

|  | **Glomerular,**  **girls** | **NG**  **non-CAKUT** | **NG CAKUT**  **low-risk** | **Reflux**  **nephropathy** | **Obstructive**  **uropathy** | **NG CAKUT high-risk other** |
| --- | --- | --- | --- | --- | --- | --- |
| Person-Visits | 477 | 855 | 961 | 635 | 886 | 360 |
| Race or ethnicity | 0% (0) | 0% (0) | 0% (0) | 0% (0) | 0% (0) | 0% (0) |
| Annual household income | 2% (25) | 1% (8) | 3% (28) | 2% (13) | 1% (10) | 1% (3) |
| Maternal education | <1% (5) | 0% (0) | 1% (9) | 2% (11) | 0% (0) | 0% (0) |
| Public health insurance | 3% (34) | 2% (18) | 3% (29) | 2% (15) | 2% (21) | 1% (5) |
| eGFR | 1% (12) | 2% (14) | 1% (8) | 1% (9) | 1% (13) | 2% (7) |
| Proteinuria | 6% (61) | 8% (68) | 11% (104) | 7% (44) | 9% (81) | 9% (32) |
| Hypertensive status | 2% (17) | 5% (42) | 5% (46) | 2% (14) | 5% (43) | 4% (15) |
| Anemia | 4% (39) | 4% (36) | 2% (24) | 4% (25) | 3% (28) | 5% (18) |
| Medication use |  |  |  |  |  |  |
| Immunosuppressant therapy | <1% (2) | 1% (5) | 1% (7) | <1%  (3) | <1%  (3) | 0% (0) |
| Corticosteroid therapy | <1% (2) | 1% (5) | 1% (7) | <1%  (3) | <1%  (3) | 0% (0) |
| Antibiotics | <1% (2) | 1% (5) | 1% (7) | <1%  (3) | <1%  (3) | 0% (0) |
| TMP-SMX | <1% (2) | 1% (5) | 1% (7) | <1%  (3) | <1%  (3) | 0% (0) |
| Diuretics | <1% (2) | 1% (5) | 1% (7) | <1%  (3) | <1%  (3) | 0% (0) |
| Bladder medication | <1% (2) | 1% (5) | 1% (7) | <1%  (3) | <1%  (3) | 0% (0) |
| Enuresis (incontinence) during the day or night | 6% (56) | 7% (56) | 6% (53) | 8% (49) | 8% (71) | 6% (22) |
| Catheterized bladder | 5% (54) | 6% (55) | 5% (49) | 7% (43) | 6% (56) | 6% (22) |

**Supplemental Table 4.** List of principal site investigators of the Chronic Kidney Disease in Children (CKiD) cohort study.

| **Study Investigator(s)** | **Institution** | **City** | **State/Province** |
| --- | --- | --- | --- |
| Kyle Deville | University of Alabama at Birmingham (Children’s of Alabama) | Birmingham | AL |
| Anjali Nayak, MD | Phoenix Children’s Hospital | Phoenix | AZ |
| Tom Blydt-Hansen, MD, FRCPC | British Columbia Children’s Hospital | Vancouver | British Columbia, Canada |
| Cynthia Wong, MD | Stanford University | Palo Alto | CA |
| Isidro Salusky, MD | University of California – Los Angeles (UCLA) | Los Angeles | CA |
| Elizabeth Ingulli, MD;  Robert Mak, MD, PhD | University of California – San Diego (UCSD) | San Diego | CA |
| Elaine Ku, MD | University of California – San Francisco (UCSF) | San Francisco | CA |
| Cheryl Sanchez-Kazi, MD | Loma Linda University | Loma Linda | CA |
| Denver Brown, MD | Children’s Research Institute  (Children’s National Medical Center) | Washington | DC |
| Sonal Bhatnagar, MD | Nemours Children’s Hospital, Delaware | Wilmington | DE |
| Marissa DeFrietas, MD; Carolyn Abitbol, MD | University of Miami | Miami | FL |
| Larry Greenbaum, MD, PhD | Children’s Healthcare of Atlanta / Emory | Atlanta | GA |
| Lyndsay Harshman, MD | University of Iowa | Iowa City | IA |
| Priya Verghese, MBBS (MD), MPH | Ann & Robert H. Lurie Children’s Hospital of Chicago | Chicago | IL |
| Sonia Krishnan, MD | University of Illinois at Chicago | Chicago | IL |
| Marciana Laster, MD | Riley Hospital for Children at Indiana University Health | Indianapolis | IN |
| Stefan Kiessling, MD; Margaret Murphy, PhD;  Aftab Chishti, MD | University of Kentucky | Lexington | KY |
| Siddharth Shah, MD | University of Louisville  (Norton Healthcare) | Louisville | KY |
| Samir El-Dahr, MD | Tulane University | New Orleans | LA |
| Nancy Rodig, MD | Boston Children’s Hospital | Boston | MA |
| Allison Dart, MD MSc, FRCPC | University of Manitoba  (Children’s Hospital Research Institute of Manitoba) | Winnipeg | Manitoba, Canada |
| Meredith Atkinson, MD;  Cozumel Pruette, MD | Johns Hopkins University School of Medicine | Baltimore | MD |
| Zubin Modi, MD | University of Michigan, Mott Hospital | Ann Arbor | MI |
| Jason Thomas, MD | Corewell Health  (Helen DeVos Children's Hospital) | Grand Rapids | MI |
| Bradley Warady, MD;  Donna Claes, MD;  Rebecca Johnson, PhD | Children’s Mercy Hospital - Kansas City | Kansas City | MO |
| Eileen Ciccia, MD | Washington University in St. Louis  (St. Louis Children’s Hospital) | St. Louis | MO |
| Stephen Hooper, PhD | University of North Carolina | Chapel Hill | NC |
| Susan Massengill, MD | Levine Children’s Hospital | Charlottesville | NC |
| Liliana Gomez-Mendez, MD | East Carolina University | Greenville | NC |
| Joann Carlson, MD | Rutgers-Robert Wood Johnson Medical School | New Brunswick | NJ |
| Craig Wong, MD, MPH | University of New Mexico Health Sciences Center | Albuquerque | NM |
| Frederick Kaskel, MD, PhD | Albert Einstein College of Medicine / Montefiore Medical Center | Bronx | NY |
| Jeffrey Saland, MD | Icahn School of Medicine at Mount Sinai | New York | NY |
| Rebecca Levy, MD | University of Rochester | Rochester | NY |
| Anil Mongia, MD | State University of New York, Downstate Medical Center | Brooklyn | NY |
| Mark Mitsnefes, MD | Cincinnati Children’s Hospital Medical Center (CCHMC) | Cincinnati | OH |
| Katherine Dell, MD | Case Western Reserve University / Cleveland Clinic Children’s | Cleveland | OH |
| Emily Stonebrook, MD | Nationwide Children’s Hospital, Ohio State University | Columbus | OH |
| Ikuyo Yamaguchi, MD, PhD | University of Oklahoma Health Sciences Center | Oklahoma City | OK |
| Kelsey Richardson, MD | Oregon Health and Science University | Portland | OR |
| Susan Furth, MD, PhD;  Erum Hartung, MD, MTR | Children’s Hospital of Philadelphia | Philadelphia | PA |
| Smitha Vidi, MD | UT Southwestern Medical Center | Dallas | TX |
| Joshua Samuels, MD | University of Texas Health Science Center at Houston | Houston | TX |
| Poyyapakkam Srivaths, MD | Baylor College of Medicine (Texas Children’s Hospital) | Houston | TX |
| Victoria Norwood, MD | University of Virginia | Charlottesville | VA |
| Joseph Flynn, MD;  Susan Halbach, MD | Seattle Children’s Hospital | Seattle | WA |
| Victoria Besser, MD | Medical College of Wisconsin | Milwaukee | WI |
| Sharon Bartosh, MD | University of Wisconsin | Madison | WI |
